# Moranoline-Enriched *Bacillus velezensis* AmoreLumina Culture Extract Attenuates Post-Inflammatory Hyperpigmentation in Acne-Prone Skin

**DOI:** 10.3390/ijms27156837

**Published:** 2026-07-30

**Authors:** Daejin Min, Hyuk Kim, Eun-Jeong Choi, Nok Hyun Park, Geunhyuk Jang, Won-Seok Park, Hyunjung Choi

**Affiliations:** Research and Innovation Center, Amorepacific Corporation, Yongin-si 17074, Gyeonggi-do, Republic of Korea; djmin@amorepacific.com (D.M.); atharabia@amorepacific.com (H.K.); ejchoi@amorepacific.com (E.-J.C.); aquareve@amorepacific.com (N.H.P.); hyuk@amorepacific.com (G.J.); wspark@amorepacific.com (W.-S.P.)

**Keywords:** PIH, macrophage, moranoline, *Bacillus velezensis* AmoreLumina, melanin

## Abstract

Post-inflammatory hyperpigmentation (PIH) results from inflammatory responses that leave persistent dark spots around pores after acne lesions are resolved. This pigmentation is caused by increased melanogenesis in melanocytes stimulated by acne-causing bacteria, followed by phagocytosis of excess melanin by macrophages that remain in the dermis. Therefore, to eliminate PIH caused by acne, it is essential not only to suppress excessive melanin synthesis by melanocytes but also to promote the degradation of melanin retained within macrophages. In this study, we investigated whether a culture extract of *Bacillus velezensis* AmoreLumina (AL), containing more than 80% moranoline (1-Deoxynojirimycin), could inhibit acne-induced pigmentation. Moranoline reduces melanin synthesis by inhibiting the glycosylation of tyrosinase, a key melanogenic enzyme, thereby suppressing its activity. The extract suppressed the upregulation of melanogenic enzymes (tyrosinase, TRP1, and TRP2) and melanin production in melanocytes exposed to acne bacteria, while promoting melanin degradation via macrophage lysosomal activity, as assessed by the relative protein expression level of p62 using Western blotting. Furthermore, in an ex vivo human skin model subjected to acne and ultraviolet radiation-induced pigmentation, treatment with the extract (0.2%) significantly reduced pigmentation (relative delta L 2.98, *p* value < 0.01). These findings suggest that cosmetic or pharmaceutical formulations incorporating AL extract or moranoline may potentially improve PIH caused by acne-related inflammation.

## 1. Introduction

Skin pigmentation is primarily regulated by melanogenesis, which is initiated when the skin is exposed to internal or external stimuli such as ultraviolet radiation (UVR), microenvironmental stiffness, inflammation, and hormones [1,2,3]. Melanin is synthesized in the melanosomes of melanocytes and subsequently transferred to surrounding keratinocytes, where it is distributed across the epidermis, resulting in skin darkening [4,5,6]. Over time, melanin within keratinocytes is naturally eliminated through desquamation, and the skin returns to its original tone. However, post-inflammatory hyperpigmentation (PIH), which results from acne, melasma, or wounds, often persists for an extended period and the skin fails to return to its original tone even after the internal and external stimuli have resolved [7,8,9,10]. This surplus melanin accumulates in keratinocytes and also infiltrates dermal macrophages recruited during inflammation, preventing complete clearance and leaving persistent pigment deposits, similar to tattoo marks [11,12,13]. Acne-related hyperpigmentation is particularly severe because melanocyte stem cells are densely localized around hair follicles, making these regions highly susceptible to inflammatory stimuli, such as *Cutibacterium acnes* (*C. acnes*) [14,15].

The skin lacks intrinsic enzymes capable of directly degrading melanin; therefore, conventional treatments for PIH rely on physical removal through keratolysis [16,17]. However, these approaches have limited effects on dermal melanin. This is because dermatological procedures that aim to eliminate melanin located in the deeper layers of the skin, such as laser therapy, simultaneously induce micro-injuries that can trigger inflammatory responses, potentially increasing the degree of PIH [18,19]. Consequently, effective management of inflammation-induced hyperpigmentation requires novel therapeutic strategies tailored to its underlying mechanisms.

Macrophages play a critical role in maintaining skin homeostasis by phagocytosing pathogens, promoting immune activation, facilitating tissue repair, and clearing senescent cells [20,21]. These functions are largely mediated by autophagy; therefore, enhancing macrophage autophagic activity could enable degradation of dermal melanin [22,23]. In this context, we aimed to investigate the potential of *Bacillus velezensis* AmoreLumina (AL) culture extract as a therapeutic approach for acne-induced inflammatory hyperpigmentation. The extract contains moranoline, which inhibits tyrosinase glycosylation and thereby suppresses melanogenesis [24,25].

## 2. Results

### 2.1. Inhibitory Effects of Moranoline-Enriched AL Culture Extract on C. acnes-Induced Melanogenesis in Normal Human Melanocytes

**Figure 1:**
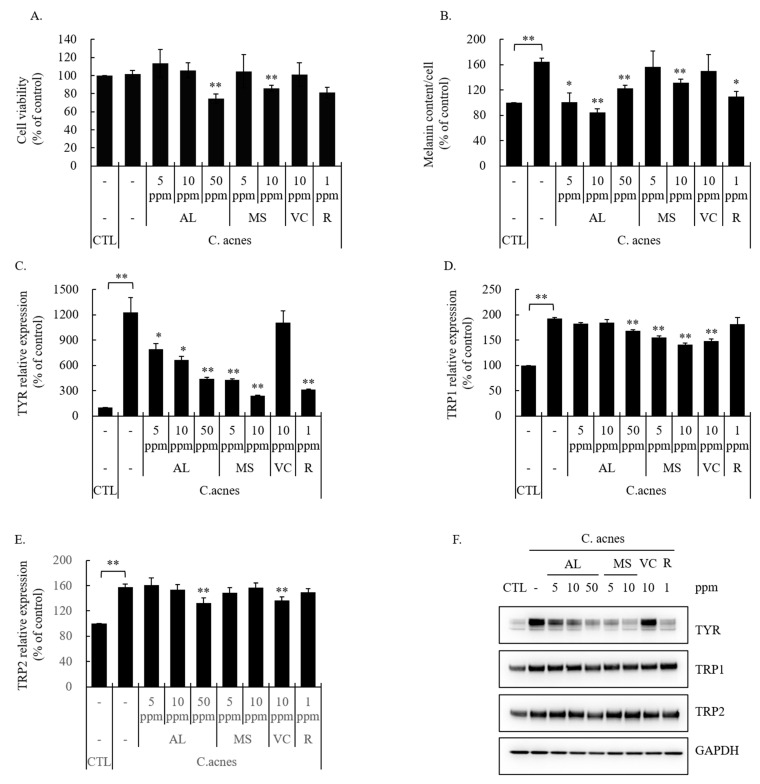
Effects of moranoline-enriched AmoreLumina (AL) culture extract on melanogenesis in normal human melanocytes (NHMs) treated with *Cutibacterium acnes*. (**A**) Viability of cells in moranoline-enriched AL culture extract (AL; 5, 10, 50 μg/mL), melasolvTM (MS; 5, 10 μg/mL), vitamin C (VC; 10 μg/mL), and 4-n-butyl-resorcinol (R; 1 μg/mL) was evaluated using Cell Counting Kit-8 (CCK-8). NHMs were treated with *C. acnes* (107 CFU/mL) and each compound at the indicated concentration for 6 days before the cell viability and melanin assays. (**B**) Melanin contents were normalized on the basis of protein concentrations to calculate the amount of melanin per cell. The relative protein expression levels of (**C**) tyrosinase (TYR), (**D**) tyrosinase-related protein 1 (TRP1), and (**E**) tyrosinase-related protein 2 (TRP2), normalized to GAPDH (loading control), were analyzed using an image analysis software for Western blotting. (**F**) Representative Western blotting data are shown. The control (CTL) was a sample from unstimulated NHMs control with *C. acnes*. The values shown represent the mean ± SD of three independent experiments (*n* = 3). * *p* ≤ 0.05 and ** *p* ≤ 0.01 vs. *C. acnes*.

To investigate acne-associated hyperpigmentation, *C. acnes* was directly applied to normal human melanocytes (NHMs). After 7 days of incubation, *C. acnes* treatment markedly increased melanin production, resulting in >50% elevation from that in untreated controls. Treatment with the moranoline-enriched AL culture extract (AL), which contained more than 80% moranoline, dose-dependently reduced the *C. acnes*-induced increase in melanin production, with melanin levels nearly restored to baseline. For comparison, the effects of well-known melanogenesis inhibitors, including vitamin C, Melasolv™, and resorcinol, were also evaluated at non-cytotoxic concentrations (Figure 1A) [26,27,28]. Under the experimental conditions, vitamin C failed to suppress the *C. acnes*-induced increase in melanin content, whereas both Melasolv™ and resorcinol significantly inhibited melanin production induced by *C. acnes* (Figure 1B). These results suggest that not all compounds known to inhibit melanogenesis may be effective against acne bacteria-induced melanogenesis. To further elucidate the molecular basis of *C. acnes*-induced melanogenesis, the protein expression levels of key melanogenic enzymes were examined using Western blot analysis (Figure 1F). Treatment with *C. acnes* significantly increased the protein expression levels of tyrosinase, tyrosinase-related protein 1 (TRP1), and tyrosinase-related protein 2 (TRP2) in NHMs. Notably, tyrosinase expression was increased by more than 10-fold following *C. acnes* exposure (Figure 1C). Consistent with their inhibitory effects on melanin production, the moranoline-enriched AL culture extract, Melasolv™, and resorcinol significantly suppressed the *C. acnes*-induced upregulation of tyrosinase expression. These findings suggest that the inhibitory effect of these agents on melanin synthesis may be closely associated with the inhibition of tyrosinase expression. In addition, unlike the other tested compounds, the moranoline-enriched AL culture extract significantly reduced the protein expression levels of TRP1 (Figure 1D) and TRP2 (Figure 1E) at 50 ppm.

Moranoline, the major component of the AL culture extract, has been reported to inhibit tyrosinase activity by suppressing its glycosylation. Therefore, in the context of *C. acnes*-induced melanogenesis, the present results further suggest that the culture extract suppresses not only tyrosinase activity but also the protein expression of key enzymes involved in melanogenesis.

### 2.2. Moranoline-Enriched AL Culture Extract Promoted Melanin Degradation in Macrophages

**Figure 2:**
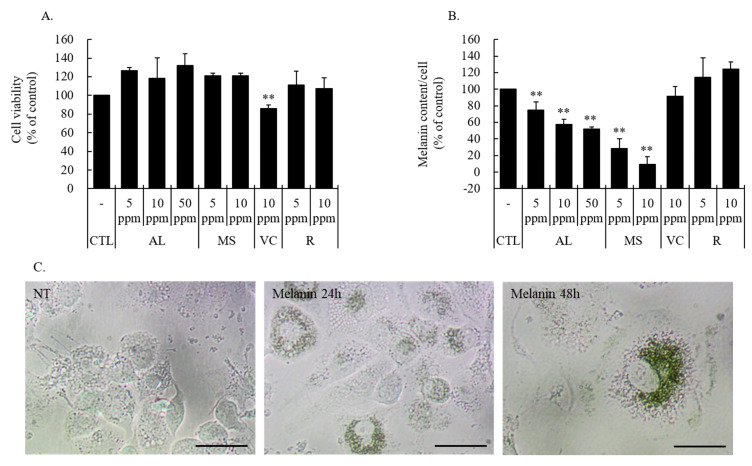

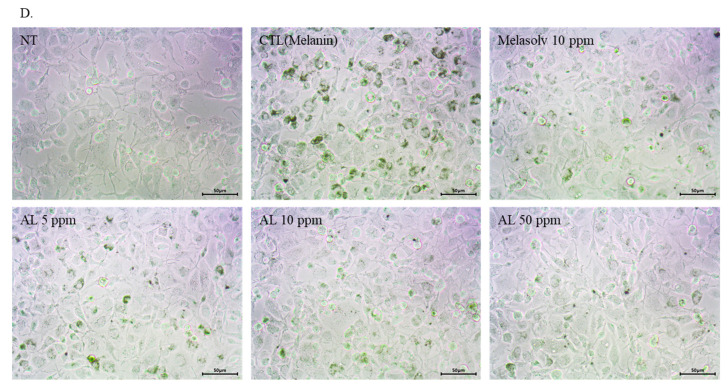
Effects of moranoline-enriched AL culture extract on melanin decomposition in macrophages. (**A**) Melanin-loaded macrophage cell viability was evaluated after treatment with moranoline-enriched AL culture extract (AL; 5, 10, and 50 µg/mL), melasolvTM (MS; 5 and 10 µg/mL), vitamin C (VC; 10 µg/mL), and 4-n-butyl-resorcinol (R; 1 µg/mL) using Cell Counting Kit-8 (CCK-8). (**B**) The melanin content of macrophages treated with each compound at the indicated concentration for 6 days was normalized on the basis of protein concentrations to calculate the amount of melanin per cell. (**C**) Representative optical microscopy images of macrophages after melanin treatment at 24 h and 48 h are shown. Scale bar = 20 µm. (**D**) Representative optical microscopy images of melanin-containing macrophages treated with varying concentrations of the compound for 6 days are shown. Scale bar = 50 µm. The CTL was a sample from melanin-containing macrophages control without compounds. The values shown represent the mean ± SD of three independent experiments (*n* = 3). * *p* ≤ 0.05 and ** *p* ≤ 0.01 vs. CTL.

Further experiments were conducted to investigate whether the moranoline-enriched AL culture extract, which suppresses *C. acnes*-induced melanogenesis in melanocytes, also affects melanin degradation in macrophages. Macrophages differentiated from THP-1 monocytes were first incubated with synthetic melanin to generate melanin-laden macrophages. Microscopic observation at 24 and 48 h after melanin treatment confirmed efficient internalization of dark melanin pigments into the cells (Figure 2C).

Subsequently, the test compounds were applied to the melanin-loaded macrophages at various concentrations, and cell viability and intracellular melanin content were assessed after 6 days of treatment. Except for vitamin C at 10 ppm, none of the tested compounds exhibited cytotoxic effects at the concentrations used (Figure 2A). Both the moranoline-enriched AL extract and Melasolv™ significantly and dose-dependently promoted melanin degradation in macrophages, resulting in a marked reduction in intracellular melanin levels compared with those in the control group (Figure 2B,D). Notably, purified moranoline exhibited a melanin-degrading effect comparable to that of the moranoline-enriched AL extract when used at an equivalent concentration. This observation suggested that moranoline may be a major contributor to the activity of the extract (Figure S1). In contrast, vitamin C and resorcinol did not enhance melanin degradation in macrophages at the tested concentrations. Melasolv™ selectively enhances melanophagy, which involves the degradation of melanosomes in melanocytes [29,30]. The present findings suggest that Melasolv™ may also promote melanin degradation in macrophages. Moreover, these results indicate that not all skin-brightening agents possess the ability to enhance melanin degradation, highlighting a clear functional distinction among skin-brightening compounds with respect to macrophage-mediated melanin clearance.

### 2.3. Moranoline-Enriched AL Culture Extract Enhanced Macrophage Melanin Degradation in Association with Autophagy/Lysosomal Pathway Markers

**Figure 3:**
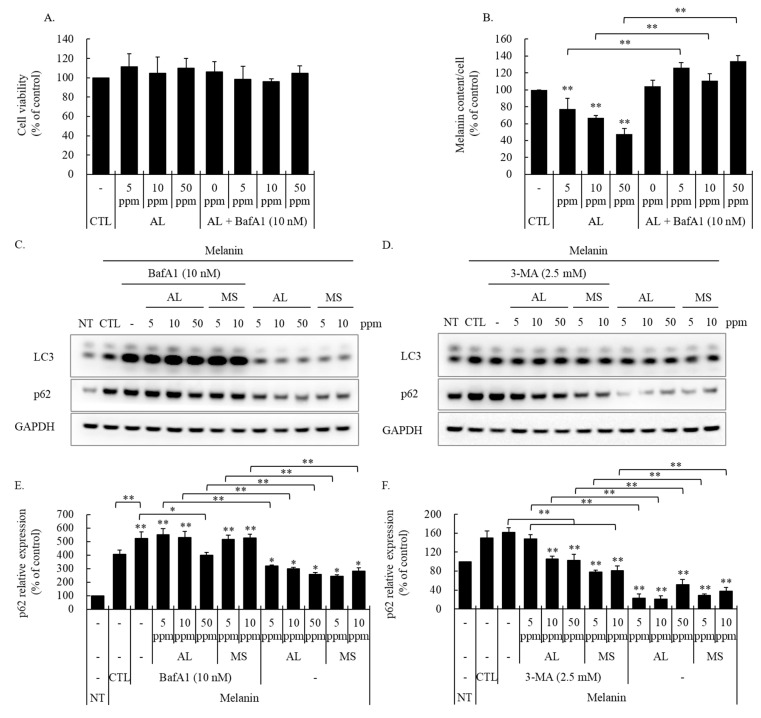
Effect of autophagy inhibitors on the melanin-degrading activity of moranoline-enriched AL culture extract. (**A**) Cell viability of macrophages treated with the moranoline-enriched AL culture extract (AL; 5, 10, and 50 μg/mL) alone or in combination with the autophagy inhibitor bafilomycin A1 (BafA1; 10 nM), assessed using the Cell Counting Kit-8 (CCK-8) assay. (**B**) Intracellular melanin content in macrophages treated with the moranoline-enriched AL culture extract alone or in combination with BafA1 (10 nM). Macrophages were treated with the culture extract for 6 days, and autophagy inhibitors were applied only during the final 24 h. (**C**) Representative Western blot images showing the protein expression levels of the autophagy markers LC3 and p62 in macrophages treated with the moranoline-enriched AL culture extract or Melasolv™, either alone or in combination with BafA1. (**D**) Representative Western blot images showing the protein expression levels of LC3 and p62 in macrophages treated with the moranoline-enriched AL culture extract or Melasolv™, either alone or in combination with the autophagy inhibitor 3-methyladenine (3-MA). (**E**,**F**) Densitometric analysis of p62 protein expression in (**C**) and (**D**), respectively, normalized to GAPDH as a loading control, using image analysis software. Data are presented as the mean ± SD of three independent experiments (*n* = 3). * *p* ≤ 0.05 and ** *p* ≤ 0.01 vs. CTL.

To determine whether the melanin-degrading activity of the moranoline-enriched AL culture extract in melanin-laden macrophages is associated with autophagy/lysosomal processing, autophagy-related inhibitors were co-administered during AL treatment. As summarized in Figure 3B, co-treatment with bafilomycin A1 (BafA1; 10 nM) abolished the dose-dependent reduction in intracellular melanin induced by AL, and BafA1 alone did not markedly alter intracellular melanin levels. Cell viability was not significantly affected in any treatment group under the conditions tested (Figure 3A). To further assess changes in autophagy-related markers associated with AL-induced melanin clearance, the protein expression of LC3 and p62 was examined using Western blotting. Compared with the control, AL treatment did not substantially change LC3 levels, but it reduced p62 protein expression. A similar pattern was observed with Melasolv™, suggesting that enhanced degradation of p62-containing cargo may accompany the melanin-degrading activity. Importantly, the AL (or Melasolv™)-associated decrease in p62 was attenuated by co-treatment with BafA1 (10 nM), consistent with the involvement of lysosomal degradation in this response (Figure 3C,E). In addition, co-treatment with another autophagy inhibitor, 3-methyladenine (3-MA; 2.5 mM), produced comparable attenuation of p62 reduction under the same experimental design shown in Figure 3D,F. Together, these inhibitor studies indicate that the melanin-degrading effect of AL in macrophages is associated with autophagy/lysosomal processing, particularly with p62-related cargo degradation and lysosomal function, rather than overt increases in LC3 abundance.

### 2.4. Inhibitory Effects of Moranoline-Enriched AL Culture Extract on Pigmentation in Human Biopsy Skin Explants

**Figure 4:**
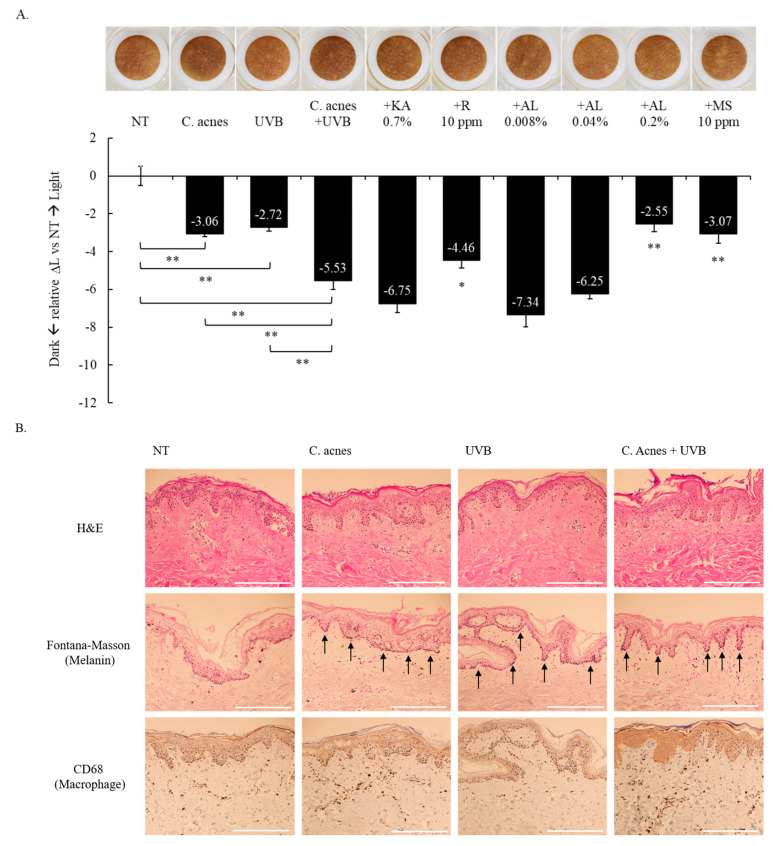

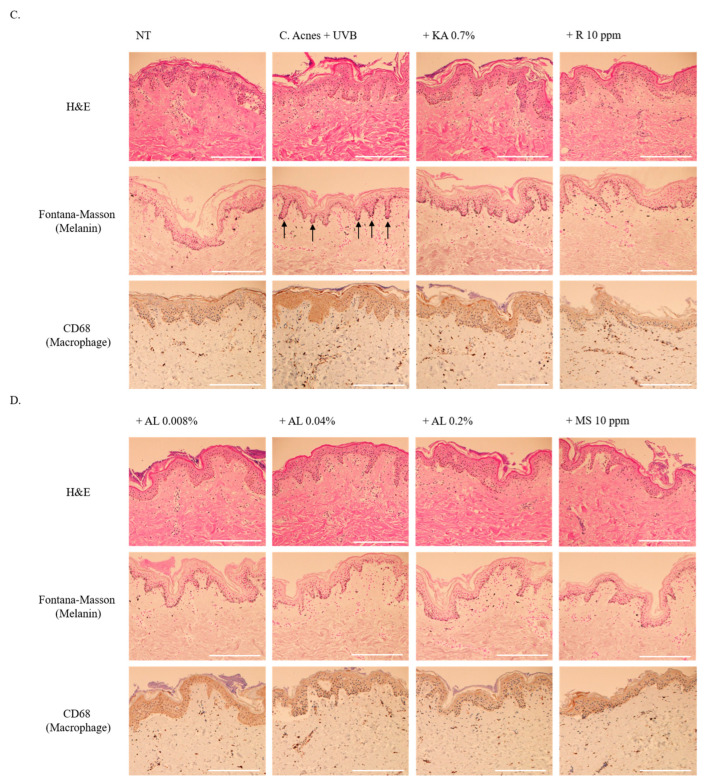
Moranoline-enriched AL culture extract suppresses *Cutibacterium acnes* + UVB-induced pigmentation in human biopsy skin explants. (**A**) Representative images of human biopsy skin explants and quantification of skin darkening expressed as relative ΔL vs. NT (L* value; lower values indicate darker skin). Explants were treated with *C. acnes* alone, UVB alone, or *C. acnes* + UVB, followed by treatment with kojic acid (KA, 0.7%), resorcinol (R, 10 ppm), moranoline-enriched AL culture extract (AL; 0.008%, 0.04%, or 0.2%), or Melasolv™ (MS, 10 ppm) as indicated. Statistical significance versus the *C. acnes* + UVB group is denoted by asterisks in the plot. (**B**–**D**) Representative histological and immunohistochemical images of biopsy skin explants following the indicated treatments. Sections were stained with hematoxylin and eosin (H&E; to visualize tissue morphology and surface bacterial coverage), Fontana-Masson (to visualize melanin deposition), and CD68 immunohistochemistry (macrophage marker). Panel sets are organized as follows: (**B**) NT, *C. acnes*, UVB, and *C. acnes* + UVB; (**C**) *C. acnes* + UVB with KA (0.7%) or R (10 ppm); (**D**) *C. acnes* + UVB with AL (0.008%), AL (0.04%), AL (0.2%), or MS (10 ppm). Arrows in Fontana-Masson images highlight increased melanin signals at the epidermal–dermal junction. Scale bar: 250 μm. Data are presented as the mean ± SD of three independent experiments (*n* = 3). * *p* ≤ 0.05 and ** *p* ≤ 0.01 vs. *C. acnes* + UVB.

To evaluate whether pigmentation could be induced in human biopsy skin explants via UVB irradiation and/or *C. acnes*, we exposed explants to UVB alone, *C. acnes* alone, or the combination of UVB plus *C. acnes*, and then quantified skin color changes by measuring the L* value before and after treatment. The change in brightness was expressed as ΔL (delta L). A decrease in the L* value corresponds to skin darkening. In this model, UVB or *C. acnes* alone induced a reduction in ΔL (skin darkening), and the combined UVB + *C. acnes* challenge produced a larger decrease in ΔL than that with either stimulus alone, indicating an additive/synergistic pigmentation-promoting effect. In a representative dataset from the same experimental framework, the relative ΔL values were decreased by single treatments and decreased further by the combined UVB + *C. acnes* condition. Next, to test the anti-pigmentation efficacy of the moranoline-enriched AL culture extract in this ex vivo setting, 0.2% of the culture extract was applied to the UVB + *C. acnes* co-treated group. Treatment with the culture extract inhibited UVB + *C. acnes*-induced darkening of the skin, indicating a suppressive effect on inflammation-associated pigmentation in human biopsy skin explants. In the ex vivo human skin pigmentation model, treatment with the extract (0.2%) significantly reduced pigmentation, with a reported relative delta L of 2.98 (*p* < 0.01). The culture extract was previously confirmed to promote macrophage-mediated melanin degradation; therefore, we also evaluated Melasolv™, which similarly enhanced macrophage melanin degradation in the same UVB + *C. acnes* model. Treatment with Melasolv™ (10 ppm) significantly inhibited UVB + *C. acnes*-induced pigmentation. In contrast, resorcinol—which suppresses melanogenesis in melanocytes without enhancing macrophage melanin degradation—also significantly reduced UVB + *C. acnes*-induced pigmentation, but its magnitude of inhibition was smaller than that observed with the moranoline-enriched culture extract (0.2%) or Melasolv™. In addition, kojic acid did not inhibit pigmentation induced by the combined UVB + *C. acnes* challenge in this model (Figure 4A). To determine how these macroscopic color changes corresponded to tissue-level alterations in the skin, histological and immunohistochemical analyses were performed after completion of the experiment. Histological examination showed that the overall epidermal and dermal architecture was preserved throughout the experimental period, without overt tissue disruption or necrosis. Unlike reconstructed skin models, biopsy skin explants retain multiple skin appendages and resident components, including immune cells, nerves, vasculature, and hair follicles. In the *C. acnes*-treated group, bacterial coverage on the skin surface was observed, and CD68-positive cells (a macrophage marker) were detected in the dermis [31,32]. Notably, when UVB and *C. acnes* were applied together, strong CD68 staining was observed in the epidermis, consistent with an enhanced inflammatory response [33]. However, the present histological analysis did not allow us to determine whether this signal originated from infiltrating inflammatory cells, resident immune cells, or keratinocytes. In parallel, increased melanin production was observed in the basal and suprabasal layers of the epidermis, particularly along the epidermal–dermal junction in the UVB-only, *C. acnes*-only, and UVB + *C. acnes* groups (Figure 4B). Consistent with the colorimetric findings, the increased melanin signal induced by the combined UVB + *C. acnes* condition was reduced in all treatment groups except the kojic acid group (Figure 4C,D). Furthermore, in the resorcinol and moranoline-enriched AL culture extract (0.2%) groups, there was a tendency toward reduced epidermal CD68 staining compared with that in the UVB + *C. acnes* group, suggesting a potential attenuation of inflammation-associated changes alongside pigmentation suppression.

## 3. Discussion

Hyperpigmentation is a heterogeneous clinical manifestation with distinct causes and mechanisms depending on the condition. Therefore, effective suppression often requires a tailored, symptom-specific solution rather than a one-size-fits-all approach. Acne-associated hyperpigmentation differs from UV-driven pigmentation in that the excessive melanin generated during acne-related inflammation is not confined to the epidermis; it can also be detected in dermal macrophages, reflecting melanin deposition and persistence in the dermal compartment. This feature implies that an effective strategy for acne-induced hyperpigmentation should not only reduce melanogenesis in melanocytes but also promote melanin clearance in dermal macrophages. This concept is aligned with the idea that enhancing macrophage autophagic activity may enable degradation of dermal melanin and thereby address inflammation-associated pigmentation more directly.

To test this rationale experimentally, we first examined whether compounds traditionally regarded as “melanogenesis inhibitors” also enhance macrophage-mediated melanin degradation. The results showed a functional separation: inhibition of melanogenesis does not necessarily translate into enhanced melanin degradation. For example, as observed with resorcinol, suppression of melanin production in melanocytes was not accompanied by an ability to promote macrophage melanin degradation. These findings suggest that melanogenesis inhibition alone may be insufficient for treating acne-associated hyperpigmentation, in which dermal melanin persistence is a key pathological feature. Notably, our human biopsy skin explant data further supported this mechanistic distinction: agents that combined melanogenesis suppression with macrophage melanin-degrading activity exerted a stronger inhibitory effect on pigmentation induced by the combined UVB and *C. acnes* challenge than did agents that primarily targeted melanogenesis.

In the ex vivo biopsy skin model, the combined UVB + *C. acnes* stimulation induced a larger decrease in L* (greater darkening) than did UVB or *C. acnes* alone, indicating that the co-challenge more robustly drives pigmentation. Under these conditions, the moranoline-enriched AL culture extract significantly suppressed pigmentation, as evidenced by a reported reduction in pigmentation with the extract at 0.2% (relative ΔL 2.98, *p* < 0.01). In the same model, Melasolv^TM^, which has previously shown to promote macrophage melanin degradation, also significantly inhibited pigmentation, whereas resorcinol significantly reduced pigmentation but with a smaller magnitude of effect than the extract or Melasolv^TM^. Collectively, these results indicate that dual-functional approaches (melanogenesis suppression + macrophage melanin clearance) may be advantageous for acne-associated hyperpigmentation, supporting the broader principle that mechanism-matched, symptom-specific interventions can be more effective.

Autophagy represents a plausible, well-known intracellular pathway for degrading large macromolecular substrates, and multiple agents have been reported to suppress pigmentation by enhancing autophagy in keratinocytes or melanocytes [34,35]. A prominent example is Melasolv^TM^, which has been studied as an enhancer of melanosome-specific autophagy (melanophagy) in melanocytes. In our study, Melasolv^TM^ also promoted melanin degradation in macrophages, suggesting that autophagy-linked processes may contribute to pigment clearance beyond melanocytes. However, our findings also highlight that not all agents associated with autophagy activation in other cell types necessarily enhance macrophage melanin degradation. Vitamin C and resveratrol, both frequently discussed in the context of cellular stress responses and autophagy-related pathways, did not promote melanin degradation in macrophages in our experimental setting [36,37]. This suggests that macrophage melanin clearance may require specific autophagy–lysosomal competencies or trafficking programs that are not uniformly engaged by “general” autophagy-associated compounds.

A further example of context dependence was observed in the ex vivo biopsy skin experiments. Kojic acid, which is clinically recognized to suppress UV-induced hyperpigmentation, did not inhibit pigmentation caused by the combined UVB + *C. acnes* challenge in our biopsy skin model, whereas it did suppress UV-induced pigmentation in biopsy skin under a UV-only condition [38]. This discrepancy suggests that the dominant drivers of pigmentation may differ by trigger context, and that efficacy against one pigmentation mechanism (e.g., UV-only melanogenesis) may not necessarily translate to efficacy against another (e.g., inflammation-coupled, acne-associated pigmentation). Together with the differential performance of resorcinol versus the moranoline-enriched AL culture extract and Melasolv^TM^, these findings further suggest that tailored solutions designed around etiology and mechanism are essential for improving clinically diverse hyperpigmentation phenotypes.

There are several limitations of the present study, which highlight future directions for research. To evaluate the degradation of melanin within macrophages, we used fragmented synthetic melanin that could be readily taken up by macrophages. In human skin, however, melanin is more likely to be transferred as melanosomes derived from melanocytes, and the physicochemical properties of melanin in melanosomes may differ from those of fragmented synthetic melanin [39,40]. Therefore, future studies should examine how melanosomes isolated from melanocytes are taken up by macrophages and whether the internalized melanosomes are degraded in response to the moranoline-enriched AL culture extract and comparator agents. Additionally, because reports suggest that macrophage differentiation status can influence melanocyte melanogenesis and differentiation, future experiments should consider macrophage phenotypic states as a variable that may shape both melanin uptake and degradation capacity [41].

Moreover, although our in vitro melanocyte experiments showed that *C. acnes* exposure increased melanin production, *C. acnes* is unlikely to penetrate deeply enough in intact skin to directly contact melanocytes in vivo. This suggests that acne-associated increases in melanogenesis are likely mediated by soluble biofactors produced within the cutaneous microenvironment, rather than direct bacteria–melanocyte interactions. Therefore, future studies should identify the mediators responsible for *C. acnes*-associated melanogenesis and define the cellular sources of these mediators. In this context, elucidating the role of keratinocytes—key orchestrators of inflammatory signaling and epidermal homeostasis—may provide critical mechanistic insight into how acne-driven inflammation propagates to melanocyte activation [42]. Improved understanding of this intercellular signaling axis could enable the development of more effective, mechanism-based interventions for acne-induced inflammatory hyperpigmentation.

Furthermore, acne-associated pigmentation in vivo is a complex and chronic process that develops following acne lesions, is often localized around follicular units, and may be exacerbated by UVR exposure. Although the human biopsy skin explant model used in this study enabled the assessment of pigmentation-related responses in human skin tissue for up to 2 weeks, it may not fully reflect the temporal, anatomical, and inflammatory complexity of acne-induced pigmentation in vivo. Thus, the present results should be interpreted as suggesting the potential activity of the tested material rather than clinical efficacy. Further clinical studies in individuals with acne-induced hyperpigmentation are needed to confirm its efficacy in a clinically relevant setting.

Finally, in this study, the involvement of autophagy in AL-associated macrophage melanin clearance was inferred from changes in autophagy-related markers and pharmacological inhibitor experiments, rather than from direct measurement of autophagic flux. Therefore, future studies using LC3-II turnover analysis, tandem fluorescent LC3 reporter assays, electron microscopy, lysosomal activity analysis, or colocalization studies with lysosomal markers are needed to determine whether the moranoline-enriched AL culture extract directly enhances autophagic flux in melanin-laden macrophages.

In summary, our results indicate that acne-associated hyperpigmentation may require a dual-target strategy that addresses both melanogenesis in melanocytes and melanin clearance in dermal macrophages. The moranoline-enriched AL culture extract significantly reduced pigmentation in an ex vivo human biopsy skin model challenged with UVB + *C. acnes*. Moreover, the comparative efficacy patterns observed across the tested agents suggest that etiology-matched, symptom-specific solutions may provide improved outcomes in complex hyperpigmentation settings.

## 4. Materials and Methods

### 4.1. Reagents

Melasolv™ (3,4,5-trimethoxycinnamate thymol ester) was synthesized by Amorepacific R&I Center (Yongin, Republic of Korea), as described previously [43]. Vitamin C (L-Ascorbic acid, A4403), 4-n-butyl-resorcinol (49797), kojic acid (K3125), BafA1 (B1793) and 3-MA (M9281) were purchased from Sigma-Aldrich (St. Louis, MO, USA).

### 4.2. Preparation of Moranoline-Enriched AL Culture Extract

The moranoline-enriched culture extract was prepared from the *Bacillus velezensis* AmoreLumina strain [44]. The strain was cultivated in a 500 mL baffled flask containing 100 mL of moranoline production medium (60 g/L sorbitol, 30 g/L soybean meal, 0.5 g/L KH_2_PO_4_, and 0.5 g/L (NH_4_)_2_SO_4_). Cultivation was performed at 37 °C with shaking at 200 rpm and an initial pH of 7.5 for 96 h. To enrich moranoline, citric acid crystals were added to the culture broth (1 L) to adjust the pH to 3.0. The broth was centrifuged at 7000 rpm for 10 min, and the supernatant was filtered through a 0.2 mm membrane. The filtrate was loaded onto a column packed with HP-20 resin (100 g, DIAION, Samyang Corp., Seoul, Republic of Korea) and eluted at a flow rate of 20 mL/min. The eluate was subsequently passed through a cation exchange column (H^+^ form, 100 g, Amberlite IR-120, Dow chemical, Midland, MI, USA) at the same flow rate, followed by washing with water (3000 mL), and elution with 0.5 N NH_4_OH. The eluate was collected in 500 mL fractions, and the first fraction showing the highest moranoline content was concentrated under reduced pressure and lyophilized. The lyophilized extract was dissolved at 5 mg/mL in acetonitrile/distilled water (50:50, *v*/*v*) containing 6.5 mM ammonium acetate (pH 5.5). Moranoline purity was analyzed using high-performance liquid chromatography (HPLC) with an amide-type column and an evaporative light-scattering detector (ELSD). Separation was performed on a TSK-Gel Amide-80 column (4.6 × 250 mm, 5 μm; Tosoh, Tokyo, Japan) with an injection volume of 10 μL, a flow rate of 1 mL/min, and a column temperature of 60 °C. The mobile phase consisted of acetonitrile/distilled water (81:19, *v*/*v*) containing 6.5 mM ammonium acetate (pH 5.5). Quantitative HPLC–ELSD analysis using an external calibration curve generated with an authentic moranoline standard (#08012; Sigma-Aldrich, St. Louis, MO, USA) showed that the resulting culture extract contained 82.3% moranoline (*w*/*w*) (Figure S2 and Tables S1 and S2).

### 4.3. Primary Melanocyte Culture and C. acnes Treatment

NHMs isolated from neonatal foreskin of moderately pigmented donors were purchased from Cascade Biologics (#C-102–5C; Portland, OR, USA). Cells were cultured in Medium 254 (#M-254–500; Cascade Biologics, Portland, OR, USA) containing Human Melanocyte Growth Supplement-2 (HMGS-2) (#S-016-5; Cascade Biologics, Portland, OR, USA). Experiments were performed using melanocytes at passages 4–7.

*C. acnes* (American Type Culture Collection [ATCC], Rockville, MD, USA) was inoculated into Brain Heart Infusion (BHI) media (Sigma, St. Louis, MO, USA) and pre-cultured for 72 h at 35–37 °C under anaerobic conditions (85% N_2_, 10% H_2_, 5% CO_2_), followed by centrifugation to collect the bacterial cells [45,46]. Then, the collected bacteria were inoculated into BHI media at a 1/100 dilution and incubated for another 72–96 h at 35–37 °C in an anaerobic environment to harvest the culture. The harvested culture was subsequently centrifuged at 4 °C, 8000× *g* for 10 min to separate the bacteria, which was then diluted in phosphate-buffered saline to 1 × 10^10^ CFU/mL and stored at −80 °C until use.

NHMs cultured for 24 h were treated with *C. acnes* and the test substance for 7 days. For this process, *C. acnes* were added to melanocytes to achieve a final concentration of 10^7^ CFU/mL. The test substance was dissolved in an appropriate vehicle (e.g., prepared at 1000× concentration in dimethyl sulfoxide and then added to the media at 1/1000 to achieve the final concentration). The same media for culturing the melanocytes were used. At the end of the cell culture, the medium was discarded, and cells were washed once with DPBS (Dulbecco’s Phosphate-Buffered Saline buffer, without calcium/magnesium chloride) followed by detachment from the culture dish using trypsin–ethylenediaminetetraacetic acid (EDTA) and collection via centrifugation.

### 4.4. Preparation of Melanin-Loaded Macrophages

The human monocytic cell line THP-1 was obtained from the ATCC (Rockville, MD, USA) and cultured at 37 °C in a humidified incubator with 5% CO_2_. Cells were maintained in RPMI 1640 medium supplemented with 10% heat-inactivated fetal bovine serum (FBS; Life Technologies, Waltham, MA, USA) and 1% penicillin/streptomycin (Life Technologies, Waltham, MA, USA). THP-1 cells were seeded at a density of approximately 1.2 × 10^6^ cells per 60 mm cell culture dish (FALCON, Corning, NY, USA). To induce macrophage differentiation, 5 mL of THP-1 culture medium containing 100 nM phorbol 12-myristate 13-acetate (PMA; Sigma-Aldrich, St. Louis, MO, USA) was added to each dish, and the cells were incubated for 48 h at 37 °C under 5% CO_2_. Differentiation into adherent macrophages was confirmed through morphological observation under a light microscope [47]. To prepare melanin-dispersed culture medium, 100 mg of synthetic melanin (M0418, Sigma-Aldrich, St. Louis, MO, USA) was added to 500 mL of THP-1 culture medium containing FBS and penicillin/streptomycin and gently mixed. The suspension was subjected to sonication (CPX8800H-E, Branson, MO, USA) for 5 min at 25 °C to achieve primary dispersion. Subsequently, the melanin suspension was further processed using a microfluidizer (M110EH-30, IDEX Material Processing Technologies, Middleborough, MA, USA) at 1500 bar for 10 passes to obtain secondary dispersion, yielding melanin particles with a size range of approximately 100–1000 nm (the reported size of intracellular melanosomes is 0.5–3 μm). The differentiated macrophages were treated with 3 mL per dish of melanin-dispersed medium and incubated for 24 h to generate melanin-containing macrophages. After incubation, intracellular melanin uptake was confirmed via light microscopy. Then, the melanin-containing medium was removed, and the cells were washed once or twice with DPBS buffer (without calcium and magnesium). Fresh differentiation medium and test compounds were administered at the indicated concentrations. The culture medium was replaced every 2 days, with fresh test compounds added at each medium change. Upon completion of treatment, cells were imaged using a light microscope, detached from the culture dishes using trypsin–EDTA and a cell scraper, and collected via centrifugation.

### 4.5. Quantification of Melanin Content and Cell Cytotoxicity Assay

To prepare cell lysates, cells were sonicated in 0.1 M Tris-HCl buffer (pH 7.2) containing 1% Nonidet P-40, 0.01% sodium dodecyl sulfate (SDS), and a protease inhibitor cocktail (Roche, Mannheim, Germany). Melanin was separated from the lysates via centrifugation at 12,000 rpm for 30 min at 4 °C and then dissolved in 1 N NaOH. The melanin content was quantified by measuring absorbance at 405 nm using an ELISA plate reader (Victor X3; PerkinElmer, Shelton, CT, USA), and the resulting optical density values were normalized to the control and expressed as percentages [48]. Cell viability was measured using the Cell Counting Kit-8 (CCK-8) assay following the manufacturer’s instructions (Dojindo Laboratories, Kumamoto, Japan). Before detaching the cells from the culture plate, culture medium containing CCK-8 diluted at a 1:100 ratio was added, and the cells were incubated at 37 °C for 1–2 h. The absorbance at 450 nm was measured, and the absolute optical density was expressed as a percentage of the control value.

### 4.6. Western Blot Analysis

Western blotting was performed using total cellular proteins extracted with RIPA lysis buffer (Sigma-Aldrich, St. Louis, MO, USA) containing protease inhibitors (Sigma-Aldrich, St. Louis, MO, USA). Following lysis, samples were centrifuged at 15,000× *g* for 10 min, and the clarified supernatants were retained. The protein content of each sample was determined using the Bradford method, with bovine serum albumin used to generate the standard curve. Protein samples (10 μg per well) were resolved via SDS-PAGE and subsequently electro-transferred to nitrocellulose membranes. The membranes were blocked in TBST buffer (10 mM Tris–HCl, pH 8.0, 150 mM NaCl, and 0.15% Tween-20) supplemented with 5% skim milk for 1 h at room temperature. Thereafter, membranes were incubated overnight at 4 °C with the following primary antibodies: anti-tyrosinase antibody (Upstate Biotechnology, Lake Placid, NY, USA), anti-TRP1 antibody (sc25543; Santa Cruz Biotechnology, Dallas, TX, USA), anti-DCT antibody (sc74439; Santa Cruz Biotechnology, Dallas, TX, USA), anti-LC3 antibody (Cell Signaling Technology, MA, USA), anti-p62 antibody (Cell Signaling Technology, Danvers, MA, USA), and anti-GAPDH antibody sc25778; Santa Cruz Biotechnology, Dallas, TX, USA). After three washes with TBST, the membranes were treated with horseradish peroxidase-conjugated goat anti-rabbit IgG secondary antibody (Bio-Rad, Hercules, CA, USA) for 1 h at room temperature. Protein bands were detected using the ECL system (Amersham Pharmacia Biotech, Piscataway, NJ, USA), and densitometric analysis of relative protein expression was carried out with ImageMaster 2D Elite software version 4.01 (Amersham Biosciences, Buckinghamshire, UK).

### 4.7. Ex Vivo Human Skin Culture

Full-thickness ex vivo human skin tissues were purchased from GENOSKIN (Toulouse, France) and cultured in the manufacturer-provided medium at 37 °C under 5% CO_2_. The human skin explants were derived from abdominal skin tissues of three female donors with Fitzpatrick skin phototype III or higher. For each donor, three independent skin specimens were prepared using an 11 mm biopsy punch and used for the explant experiments. PIH-like pigmentation was induced by co-treatment of skin tissues with *C. acnes* (1 × 10^7^ CFU/tissue) and UVB irradiation (100 mJ/cm^2^). Test compounds were administered to the culture medium at the indicated concentrations every 2 days for a total of 14 days. Skin brightness (L value) was determined by analyzing digital images of each tissue using Image-Pro^®^ Plus (ver. 4.5.0.29, Media Cybernetics, Inc., Rockville, MD, USA) and Convert RGB to Lab web software (ColorMine.org, http://colormine.org/convert/rgb-to-lab, accessed on 29 June 2026). Changes in skin brightness (relative ΔL) were calculated by subtracting the initial brightness (Li) from the final brightness (Lf). After treatment, tissues were fixed in formalin and embedded in paraffin. Sections (3 µm) were stained with hematoxylin and eosin and Fontana-Masson stain, and immunohistochemistry was performed using an anti-CD68 antibody (Kp-1, CELL MARQUE™, Rocklin, CA, USA). Stained sections were examined under a microscope.

### 4.8. Statistical Analysis

Statistical analyses of the data were performed using RStudio version 2025.05.1 (Posit PBC, Boston, MA, USA). Results are reported as the mean ± standard deviation (SD) based on three independent experimental replicates. Differences among groups were evaluated using one-way analysis of variance, with post hoc Welch two sample *t*-tests. Values of * *p* ≤ 0.05 and ** *p* ≤ 0.01 were considered statistically significant.

## 5. Conclusions

The findings of this study suggest that acne-associated hyperpigmentation may require etiology-matched solutions that address both melanogenesis in melanocytes and pigment persistence beyond the epidermis. In NHMs, *C. acnes* increased melanogenesis and upregulated melanogenic enzymes, and the moranoline-enriched AL culture extract suppressed these responses, suggesting potential activity of the culture extract against acne bacteria-associated melanogenic stimulation. In melanin-laden macrophages, AL promoted melanin degradation. This effect was associated with autophagy–lysosomal processing, as co-treatment with BafA1 (10 nM) abolished AL-induced melanin reduction and attenuated the AL-associated decrease in p62. In an ex vivo human biopsy skin explant model, the combined UVB + *C. acnes* challenge produced stronger darkening than that with either stimulus alone, and AL (0.2%) significantly suppressed pigmentation (reported relative ΔL 2.98, *p* < 0.01), while kojic acid did not inhibit pigmentation induced by the combined challenge. Collectively, these findings indicate that skin brightening agents may differ in their functional profiles, and that integrating melanogenesis inhibition with macrophage-associated pigment clearance may provide enhanced efficacy for acne-relevant inflammatory pigmentation.

## Data Availability

The data analyzed in this study are available on request from the corresponding author.

## Supplementary Materials

The following supporting information can be downloaded at https://www.mdpi.com/article/10.3390/ijms27156837/s1.

## Abbreviations

The following abbreviations are used in this manuscript:

PIH	post-inflammatory hyperpigmentation
AL	*Bacillus velezensis* AmoreLumina
UVR	ultraviolet radiation
*C. acnes*	*Cutibacterium acnes*
NHMs	normal human melanocytes
TRP1	tyrosinase-related protein 1
TRP2	tyrosinase-related protein 2
BafA1	bafilomycin A1
3-MA	3-methyladenine
HPLC	high-performance liquid chromatography
ELSD	evaporative light-scattering detector
ATCC	American Type Culture Collection
BHI	Brain Heart Infusion
PMA	phorbol 12-myristate 13-acetate
SDS	sodium dodecyl sulfate
EDTA	ethylenediaminetetraacetic acid
