# Supplementary material for: Moranoline-Enriched Bacillus velezensis AmoreLumina Culture Extract Attenuates Post-Inflammatory Hyperpigmentation in Acne-Prone Skin
Source: Int J Mol Sci. 2026 Jul 30;27(15):6837. doi: 10.3390/ijms27156837 (PMC13466432; doi:10.3390/ijms27156837)
Supplement: Supplementary file 1 [file ijms-27-06837-s001.zip › ijms-4438088-supplementary.pdf]

Supplementary data

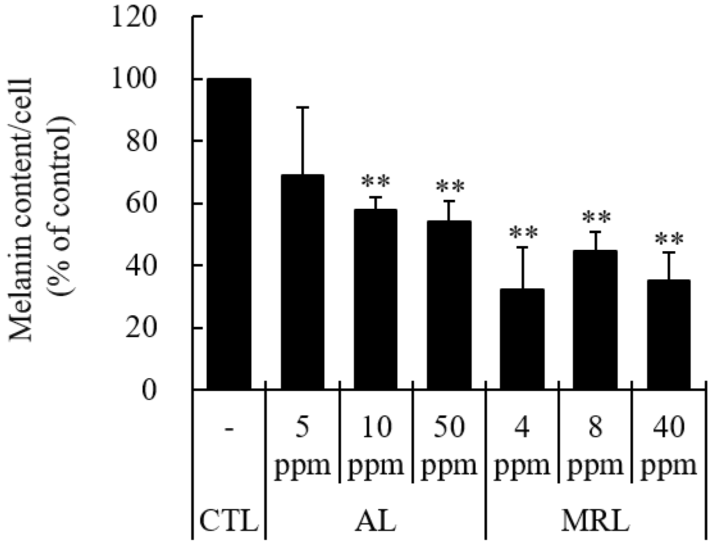

Figure S1. Effects of moranoline-enriched AL culture extract and moranoline on melanin decomposition in macrophages.

The melanin content of macrophages treated with each compound at the indicated concentration (AL, moranoline-enriched AL culture extract; MRL, moranoline from Sigma-Aldrich, Cat. No. 08012) for 6 days was normalized on the basis of protein concentrations to calculate the amount of melanin per cell. The CTL was a sample from melanin-containing macrophages control without compounds. Data are presented as the mean ± SD of triplicate wells from a single experiment. ** $P \leq 0.01$ vs. CTL.

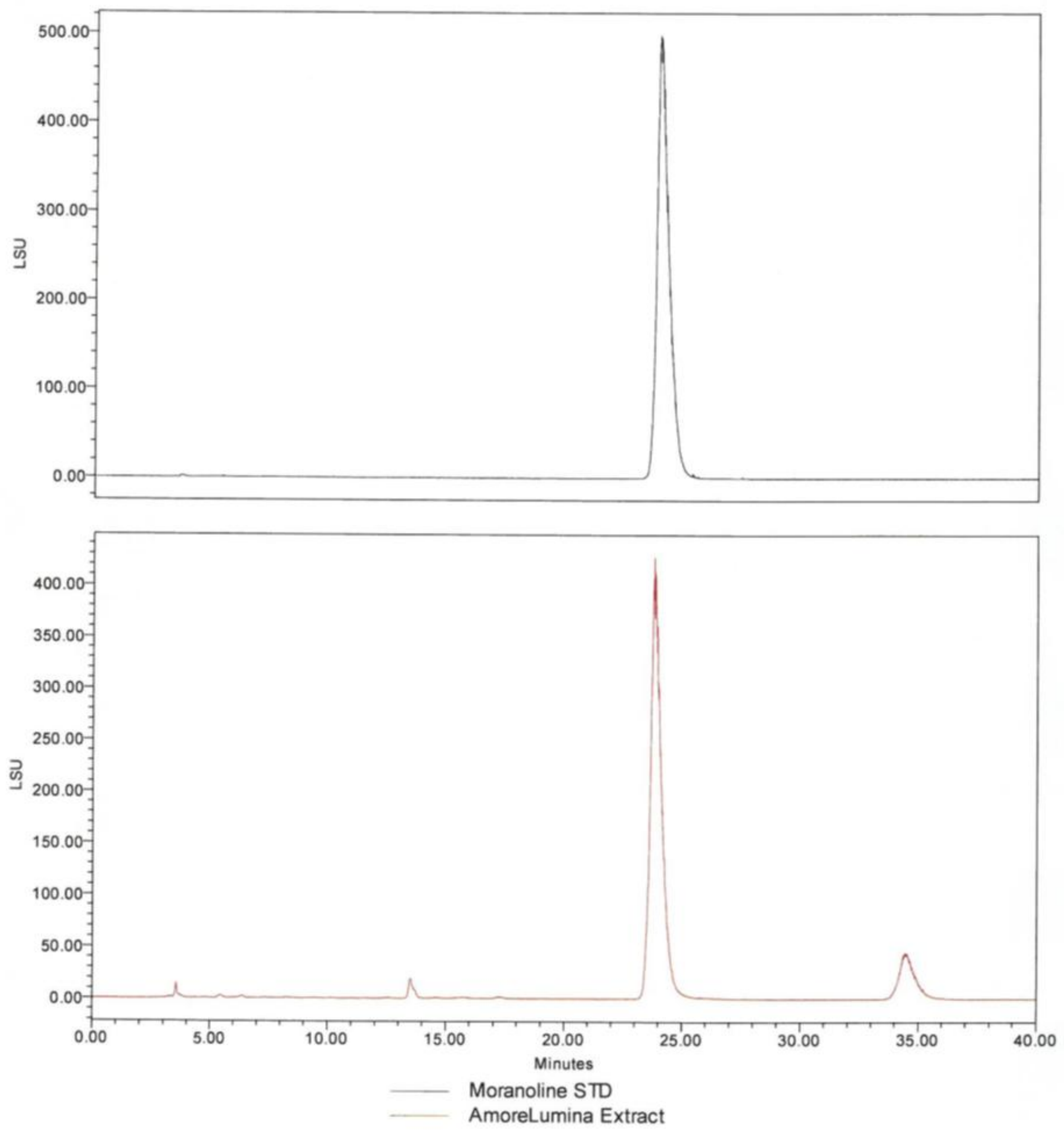

Figure S2. HPLC–ELSD analysis of moranoline in the AL culture extract. Overlay chromatograms of authentic moranoline standard (Sigma-Aldrich, Cat. No. 08012) and the AL culture extract analyzed using a TSK-Gel Amide-80 column coupled with an ELSD. The major peak detected in the culture extract exhibited the same retention time as that of the moranoline standard, confirming the presence of moranoline as the major component of the extract. HPLC, high-performance liquid chromatography; ELSD, evaporative light-scattering detector.

Table S1. Calibration data for moranoline standard used for HPLC–ELSD quantification

Moranoline standard (mg/mL)	Peak area
0.5	1,295,628
1	3,544,241
2.5	8,245,111
5	15,801,655

Authentic moranoline standard (1-deoxynojirimycin; Sigma-Aldrich, Cat. No. 08012) was used to generate an external calibration curve for quantitative HPLC–ELSD analysis.
HPLC, high-performance liquid chromatography; ELSD, evaporative light-scattering detector.

Table S2. Quantitative determination of moranoline content in the AL culture extract

Sample	Extract concentration (mg/mL)	Measured moranoline concentration (mg/mL)	Moranoline content (%)
AL culture extract	5	4.115	82.3

Moranoline concentration was determined via HPLC–ELSD using an external calibration curve generated with an authentic moranoline standard. Quantitative analysis showed that moranoline accounted for 82.3% (w/w) of the lyophilized AL culture extract.
HPLC, high-performance liquid chromatography; ELSD, evaporative light-scattering detector.
